# Optimising pre‐operative imaging–surgery intervals for stones

**DOI:** 10.1002/bco2.232

**Published:** 2023-06-12

**Authors:** Ijeoma Chibuzo, Aleksandar Vucicevic, Abisola Oliyide, Adebanji Adeyoju, Zara Gall

**Affiliations:** ^1^ Department of Urology Stepping Hill Hospital Stockport UK

**Keywords:** imaging–surgery interval, negative surgeries, reference images, upper tract stones

## Abstract

**Introduction and objectives:**

The NICE guidelines for acute ureteric colic recommend diagnostic imaging, definitive management and definitive care within 24 and 48 h of symptoms and 4 weeks of temporisation, respectively. However, the NHS reality is fraught with long waiting times to definitive treatment, further compounded by a progressively increasing stone burden, paucity of on‐site lithotripters and a decrease in non‐cancer elective theatre sessions during the COVID‐19 pandemic. By the time patients attended the elective surgeries, their reference images (RIs) were often significantly out of date. Scant direction exists on what interval between imaging and surgery invalidates the usefulness of the RIs in providing surgical guidance.

This study aimed to evaluate the role of imaging–surgery intervals (ISIs) on upper tract stone negative surgery outcomes and derive a cut‐off ISI warranting updated images, with a view to improving efficiency and patient safety.

**Materials and methods:**

Upper tract stone surgeries were retrospectively assessed. Each renal unit was considered independently in bilateral stones. Cases were grouped into renal/pelvic (referred to as ‘RENAL’) and URETERIC stones. Data retrieved included the ISI, intra‐operative disparity (IOD) between stone‐related features on RIs and the surgical findings. Receiver operating curves (ROCs) were used to determine ISI cut‐offs more predictive of IODs.

**Results:**

Four hundred and twenty‐seven surgeries on 174 (40.7%) RENAL and 253 (59.3%) URETERIC stones were appraised. No stones were found intraoperatively in 52 (12.1%) patients. Longer ISIs were associated with IODs, especially with URETERIC stones (*p* = 0.011, CI_95_ 0.63; 4.84). The derived ROC ISI cut‐offs beyond which IODs, including negative surgeries, were more likely were 9 weeks for URETERIC (AUC: 63%, CI_95_ 0.56; 0.70) and 19 weeks (AUC: 58.6%, CI_95_ 0.50; 0.68) for RENAL stones, respectively.

**Conclusion:**

There is a need to update reference imaging done more than 9 or 19 weeks before surgery for URETERIC and RENAL stones, respectively.

## 1 INTRODUCTION

Hospital Episodes Statistics has shown a rising trend in the prevalence of urinary tract stones and admissions related to urolithiasis in England.^1^ A 12%–35% rise in consultations and a 47%–79% rise in surgeries for urolithiasis across different age groups above 15 years of age were reported.^1^ The increasing burden of patients with symptomatic stones, rising lifetime prevalence of stone disease and shortage in many centres of on‐site shock wave lithotripters have led to long waiting surgical lists for patients under the National Health Service (NHS).^2^

In 2020, the impact of the COVID‐19 pandemic transcended all aspects of healthcare, modifying practice and the routine or emergent approach to management of other diseases. Guidelines recommended delaying definitive care for non‐obstructing stones and encouraged conservative options or temporisation.^3^, ^4^ An online survey by the European Section of Urolithiasis (EULIS) Collaborative group showed that 89.4% of practitioners across 20 European countries consequently changed their practice to decompress obstructed urinary systems and delay definitive treatment.^5^ Thus, the already long NHS waiting lists for stone treatment became longer.^6^

The British Association of Urology recommends definitive treatment should not exceed 4 weeks from the temporising intervention, the former having been within 48 h of diagnosis.^7^ The NICE guidelines recommend offering surgery within 48 h of diagnosis or readmission.^2^ Given the aforementioned constraints, these timelines were not being met. Further, the NHS maximum waiting times from referral to definitive treatment of 18 weeks were being exceeded.^2^, ^8^

At our centre, from March 2020 of the COVID‐19 pandemic, surgical priority was given to cancer‐related surgeries with limited elective slots for stones. Our practice changed to aim towards primary ureteroscopy for new stone diagnoses presenting as emergencies. For elective surgeries, on the other hand, we experienced incidents whereby patients had outdated reference images by the time a date for surgery was available, with discrepant findings at surgery, negative surgery (NS) or even presentation with contralateral pain, rather than pain on the side the patient was listed to have surgery on. The latter scenario resulted in change of the intended side to be operated on the day of surgery after urgent re‐imaging.

Our protocol by 2020 did not include repeat imaging on the morning of surgery as is the standard of care in some other centres.^9^ An important consideration is the possible challenge of obtaining the CT scans on the day of surgery. A request for repeat imaging at our centre is rather driven by clinical information received prior to surgery, which might indicate a need. Another consideration for same‐day pre‐operative imaging is the possible surgery cancellation(s) on the day, with no allowance for substitution of the patients, as the protocol during the pandemic involved a 2‐week pre‐operative isolation period and negative COVID swab from 3 days prior to surgery. Therefore, the precious elective slot would be wasted if a CT scan on the day of surgery showed there was no need for surgical intervention. Overall, there was limited existent evidence on the most appropriate time for repeat imaging prior to surgery.

The desire to improve the efficiency of the entire process and still ensure patient safety led to this study. We aimed to define a time limit from the last imaging beyond which patients were more likely to have surgical findings inconsistent with the prior radiological findings, or even negative surgeries, and should therefore, have updated imaging for stones.

## 2 MATERIALS AND METHODS

A retrospective review of surgeries (ureteroscopies, standard and mini‐percutaneous nephrolithotomies) done for urinary tract stones between December 2017 and November 2019 was conducted. Each urinary system was considered independently in patients with bilateral stone burden requiring surgical intervention. The cases were analysed as a cohort and dichotomised into those in the kidney or renal pelvis, hereby referred to as RENAL stones, and those between the proximal ureter and vesico‐ureteric junction (VUJ), hereby termed URETERIC stones.

Data on stone size, location, radiological and surgical findings were retrieved. The primary outcomes were the time in weeks between the reference imaging used to plan the surgery and the surgery date, termed the *imaging–surgery interval* (ISI), and discrepancy rate between pre‐operative imaging and intraoperative surgical findings, *intra‐operative disparity* (IOD). Secondary outcomes included predictors of IODs and determination of the role, if any, of ISIs in IODs. Patients with incomplete data, anatomic variations, concomitant non‐stone‐related surgeries or extracorporeal shock wave lithotripsy during the ISI were excluded from the analysis.

Measures of association were used to determine relevant factors predicting IODs. Receiver operating curves (ROCs) were used to derive ISI cut‐offs predictive of IODs.

## 3 RESULTS

Four hundred and twenty‐seven urological surgeries were analysed, comprising 174 (40.7%) RENAL and 253 (59.3%) URETERIC stones. The patients were aged 17–96, with mean age of 56 years, SD 15.7. Two hundred and sixty‐six (62.3%) were males, whereas 161 (37.7%) were female. Thirty‐seven were prescribed Tamsulosin, whereas 89 had pre‐operative stents inserted. Thirty‐two (7.5%) reference images were X‐rays, whereas the rest were non‐contrast helical CT scans. Tables 1 and 2 summarise the stone and imaging characteristics.

**TABLE 1:** Summary of ISI, stone characteristics and intra‐operative findings.

Stone location	Median ISI (range in weeks)	Median stone size in mm (range)	Intra‐operative change, n (%)			
			Any change	Number	Position	Surgery
All (*N* = 427)	11.2 (0–61.7)	8 (1–91)	171 (40.0)	117 (27.4)	114 (26.7)	44 (10.30)
RENAL (*n* = 174)	17.7 (0–61.7)	11 (2.5–91)	59 (33.9)	45 (25.9)	27 (15.5)	18 (10.34)
URETERIC (*n* = 253)	8.9 (0–44.9)	7 (1–19)	112 (44.3)	72 (28.5)	87 (34.3)	26 (10.27)

**TABLE 2:** Stone locations in all patients, those with IODs and those with NS.

SITE		All patients	IOD present	NS
		Frequency (%)	Frequency (%)	Frequency (%)
Renal	**RENAL**	102 (23.9)	39 (22.8)	4 (7.7)
Pelvis		72 (16.9)	20 (11.7)	4 (7.7)
Proximal ureter	**URETERIC**	87 (20.4)	40 (23.4)	7 (13.5)
Mid‐ureter		49 (11.5)	24 (14.0)	9 (17.3)
Distal ureter		90 (21.1)	37 (21.6)	21 (40.4)
VUJ		27 (6.3)	11 (6.4)	7 (13.5)
Total		427	171	52

Abbreviations: IOD, intra‐operative disparities; NS, negative surgeries.

Forty percent had disparate intraoperative findings when compared with the findings in the reference image. These IODs were in stone number (117, 27.4%) and position (114, 26.7%), necessitating an alteration in surgical plan (46, 10.3%) or unplanned additional procedure (14, 2.1%). The surgeries done in those with IODs included standard (15, 8.8%) and miniaturised PCNLs (18, 8.2%), combined PCNL with URS (2, 1.2%) and cystolitholapaxy for significant stent encrustation (1, 0.6%). Longer ISIs were associated with IODs, *p* = 0.11, CI_95_ 0.64; 0.484.

Fifty‐two patients (12.1%), 17 females and 35 males, had no stones at surgery. Eight (15.4%) were RENAL, whereas 44 (84.6%) were URETERIC, with 7.7%, 7.7%, 13.5%, 17.3%, 40.4% and 13.5% located in the kidney, renal pelvis, proximal, middle and distal ureter, and VUJ respectively. The mean ISI was 23.12 (SD, 10.48) in the RENAL group and 13.13 (SD, 7.5 weeks) in the URETERIC group. The mean stone size was 5.7 (SD, 2.5) mm. Twenty‐five of those with NS were right‐sided. Longer ISIs were associated with higher risk of NS, *p* = 0.028 and 0.001, CI_95_–5.47; −0.31. Pre‐operative stents had been inserted in 19 (36.5%) of those with NS. Stents were significantly associated with change in ureteric stone position, an IOD (*p* = 0.018, OR 2.1) but not NS. There was no association found between gender or laterality and NS.

With URETERIC stones, NS risk increased with longer ISI (*p* = 0.041; CI_95_ –5.6; –50.12). For RENAL stones, although NS increased with longer ISIs, this was not statistically significant, *p* = 0.437, CI_95_ –14.1; 6.12. For the entire cohort, ISI (*p* = 0.007, CI_95_ –0.011; −0.002) and dichotomised stone location (*p* = 0.001, CI_95_ –0.274; −0.068) were independent predictors of IODs, [*F*(2, 412) = 6.509, *p* = 0.002] with *R* ^2^ of 0.031 (CI_95_–0.11, −0.002). Readmissions with post‐operative Clavien–Dindo I and II complications were seen in 3.8% of those with NS.

The median URETERIC stone size was 6.5 mm (1–19 mm) with median ISI of 8.8 (0–44.9) weeks. The AUC for the ROC was 62.8% (*p* = 0.001, CI_95_ 0.558; 0.698). Identified ISI cut‐off was 8.93 weeks with a sensitivity of 62.7% and specificity of 61.9%.

For RENAL stones, the median stone size and ISI were 11 (2.5–91) mm and 17.7 (0–61.7) weeks, respectively. The AUC of the ROC was 58.6% (*p* = 0.068, CI_95_ 0.495; 0.678). The identified ISI cut‐off was 19.21 weeks with sensitivity and specificity of 60.7% each. ISI effect on stone position with RENAL stones (*p* = 0.11) was demonstrated, but not on NS (*p* = 0.783).

**FIGURE 1:**
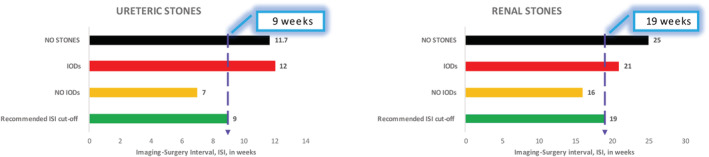
ISI cut‐offs and the relationship to intra‐operative findings, IODs.

For either group, the ISIs of those with IODs or NS, exceeded the derived ISI cut‐offs, whereas those without IODs fell within the ISI cut‐off limits (Figure 1).

## 4 DISCUSSION

The elective pathway in the NHS includes an 18‐week target for a Finished Consultant Episode and clearly states that no patient should be waiting up to a year for definitive care.^10^ The ISI ranges in this study show that these targets were not met. The pre‐pandemic backlogs were significant, and the demand for more slots exceeded capacity in the NHS.^1^ With the COVID‐19 pandemic, the backlog of cases in the community and on waiting lists awaiting definitive treatment under the NHS increased significantly, with over 380 000 having waited for over a year by April 2021.^6^, ^11^ Renal stones and non‐acute presentations were labelled non‐urgent and deferred during the peak of the pandemic in accordance with the recommendations of the rapid reaction group.^3^

A consensus on when to update the reference image used to make the diagnosis and plan for surgery is lacking at the moment. Long delays have been noted to increase the incidence of NS.^12^ It is generally agreed that negative surgeries for stones should be avoided.^12^ The CROES study^13^ demonstrated that URS is not innocuous, with up to 1.2% of all patients and 17.7% of patients with complications following URS having Clavien–Dindo Class III and IV complications. A study from the Sheffield Teaching Hospital, which had a 14% NS rate, performed pre‐operative CT scans on all patients on the day of surgery.^9^ Where consideration for cumulative radiation exposure is given, low‐dose CTs or plain X‐rays are done.^12^ Logistic constraints may not permit these image updates on the day of surgery in some centres. Even if on‐the‐day CT is available, there is a potential downside of underutilisation of the list as it can be difficult to fill the slot at short notice. This risk of not being able to fill slots at short notice was more significant during the COVID pandemic, during which elective patients isolated for 2 weeks pre‐operatively and needed to have proof of a negative ELISA test within 72 h of surgery.

The findings of our study indicate that images older than 9 weeks for URETERIC and 19 weeks for RENAL stones should be repeated as they had a higher risk of IODs, including NS. With the RENAL stones, positional change was more apparent than change in number or negative surgeries. This is intuitive, due to the lack of the displacing effect of ureteric peristalsis on the stone. Kreshover et al.^14^ evaluated predictors of NS. ISI was not found to be relevant; however, their median ISI was much shorter (40 days) compared with ours, the highest of which was 67 weeks. Interestingly, despite the non‐statistical significance in that study, those with NSs had longer ISIs.

Factors documented to affect NS rate include smaller and more distal stone size and female gender. In females, phleboliths may be mistaken for stones, increasing the apparent NS rate.^12^, ^14^ We did not find female gender important in predicting NS. This may be explained by uroradiologist involvement in CT reports at our centre, the availability of a stone MDT and less tendency for these uroradiology subspecialists to report phleboliths as stones. Our findings add ISI to the list of factors, making it the only modifiable risk factor within the control of the surgeon to vary NS outcomes.

A greater relevance of the ISI to URETERIC stones, and higher rate of NS amongst URETERIC stones (84.6%), especially the distal ureteric ones, was demonstrated in our study. This is in keeping with the information from the MIMIC^15^ and SUSPEND^16^ studies, which showed that 74%–81% of patients with ureteric stones, and 83% with distal ureteric stones, would pass their stones spontaneously if given the time to do so, thus further reiterating the dynamic nature of urolithiasis and need for updating the reference images.

## 5 LIMITATIONS

Our study was small in comparison with multicentre studies or systematic reviews and would need larger numbers and further validation to verify its findings.

It also did not define how close to the date of surgery the task of updating images should be done. We, however, recommend updating the images 1–2 weeks before the surgery to permit replacement of the patient if the surgery is no longer required by the index patient.

## 6 CONCLUSION

Increased intra‐operative disparities and negative surgeries occur with ISIs greater than 9 and 19 weeks for URETERIC and RENAL stones, respectively. Updating the reference images that have exceeded these cut‐offs could potentially reduce the risk of disparate findings at surgery, or even the risk of a stoneless surgery.

This would potentially reduce the risk to patient safety posed by an unnecessary surgery and anaesthetic exposure, as well as complications from the procedure itself. It would also allow better utilisation of theatre lists, improving efficiency, that is essential to reducing the backlog. It is also easy enough to effect.

## 7 FUTURE RESEARCH

An in‐hospital guidance to update the pre‐operative reference images using these ISI cut‐offs as a benchmark is being implemented at our centre. Further research to evaluate the incidence of NS and IODs thereafter would be necessary to validate the efficacy and accuracy of these ISI cut‐offs.

## AUTHOR CONTRIBUTIONS

The study concept, protocol design, data analysis and manuscript preparation were by Ijeoma Chibuzo; Ijeoma Chibuzo and Aleksandar Vucicevic collated the data; Abisola Oliyide, Adebanji Adeyoju and Zara Gall edited the manuscript; Adebanji Adeyoju and Zara Gall supervised the study.

## CONFLICT OF INTEREST STATEMENT

The authors have no conflicts of interest to declare.
